# An Introduction to Next Generation Sequencing Bioinformatic Analysis in Gut Microbiome Studies

**DOI:** 10.3390/biom11040530

**Published:** 2021-04-02

**Authors:** Bei Gao, Liang Chi, Yixin Zhu, Xiaochun Shi, Pengcheng Tu, Bing Li, Jun Yin, Nan Gao, Weishou Shen, Bernd Schnabl

**Affiliations:** 1 Department of Marine Science, School of Marine Sciences, Nanjing University of Information Science and Technology, Nanjing 210044, China; wintergb@hotmail.com; 2 Metaorganism Immunity Section, Laboratory of Immune Systems Biology, National Institute of Allergy and Infectious Diseases, National Institutes of Health, Bethesda, MD 20892, USA; chil2@nih.gov; 3 Department of Medicine, University of California San Diego, La Jolla, CA 92093, USA; y3zhu@ucsd.edu; 4 Department of Environmental Ecological Engineering, School of Environmental Science and Engineering, Nanjing University of Information Science and Technology, Nanjing 210044, China; 20201248131@nuist.edu.cn (X.S.); wsshen@nuist.edu.cn (W.S.); 5 Department of Food Science and Nutrition, College of Biosystems Engineering and Food Science, Zhejiang University, Hangzhou 310058, China; tupengcheng1@163.com; 6 Suzhou Industrial Park Environmental Law Enforcement Brigade (Environmental Monitoring Station), Suzhou 215021, China; lbing@sipac.gov.cn; 7 Department of Hydrometeorology, School of Hydrology and Water Resources, Nanjing University of Information Science and Technology, Nanjing 210044, China; jy.junyin@foxmail.com; 8 Department of Biotechnology, School of Biological and Pharmaceutical Engineering, Nanjing Tech University, Nanjing 211816, China; ngao@njtech.edu.cn; 9 Jiangsu Key Laboratory of Atmospheric Environment Monitoring and Pollution Control, Collaborative Innovation Center of Atmospheric Environment and Equipment Technology, Nanjing 210044, China; 10 Department of Medicine, VA San Diego Healthcare System, San Diego, CA 92161, USA

**Keywords:** gut microbiota, fungi, virus

## Abstract

The gut microbiome is a microbial ecosystem which expresses 100 times more genes than the human host and plays an essential role in human health and disease pathogenesis. Since most intestinal microbial species are difficult to culture, next generation sequencing technologies have been widely applied to study the gut microbiome, including 16S rRNA, 18S rRNA, internal transcribed spacer (ITS) sequencing, shotgun metagenomic sequencing, metatranscriptomic sequencing and viromic sequencing. Various software tools were developed to analyze different sequencing data. In this review, we summarize commonly used computational tools for gut microbiome data analysis, which extended our understanding of the gut microbiome in health and diseases.

## 1. Introduction

The gut microbiome is a complex ecosystem with great impacts on the overall health of the host [1,2,3]. These microorganisms living in the gastrointestinal tract have various functionalities, such as absorption of nutrients and minerals, fermentation of fibers to short-chain fatty acids, synthesis of vitamins, breakdown of toxic components, and regulation of the immune system. The gut microbiome changes over time depending on host’s age and dietary habits [4]. Its status is in close correlation to many diseases such as liver diseases [5,6,7], diabetes [8], inflammatory bowel disease [9,10], autoimmune diseases [11,12], colorectal cancer [13] and diseases of the central nervous system [14].

**Figure 1:**
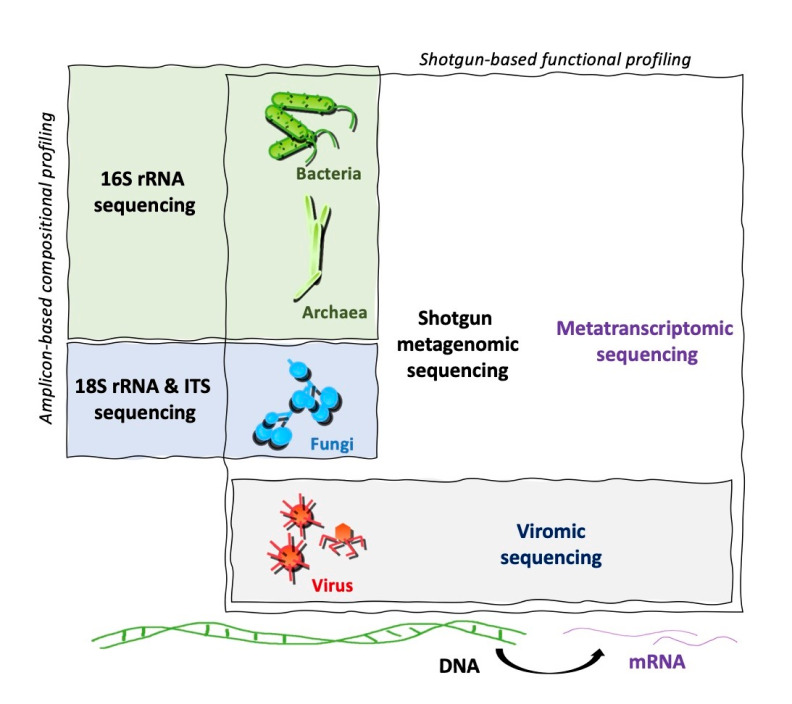
Commonly used sequencing techniques for the gut microbiome study.

Widely used high-throughput sequencing methods in microbiome research include PCR amplicon-based sequencing, e.g.,16S rRNA, 18S rRNA, internal transcribed spacer (ITS) sequencing, DNA-based shotgun metagenomic sequencing, RNA-based metatranscriptomic sequencing, and viromic sequencing (Figure 1). The first decade of gut microbiome research has mainly focused on DNA-based 16S rRNA gene sequencing and shotgun metagenomic sequencing, which elucidate the microbial composition and gene content. Recently, more attention has been drawn on RNA-based approach, metatranscriptomic sequencing, as well as on fungi and viruses, instead of solely focusing on bacteria. Various computational techniques have been developed to analyze different types of high-throughput sequencing data. The best practice for performing a microbiome study has been reviewed by Knight et al., including experiment design, choice of molecular analysis technology, etc. [15]. In this review, we will summarize commonly used computational tools used for the analysis of different types of sequencing data in the gut microbiome studies, which help to extend our knowledge in the role gut microbiome plays in human health and disease pathogenesis.

## 2. 16S rRNA Sequencing

**Figure 2:**
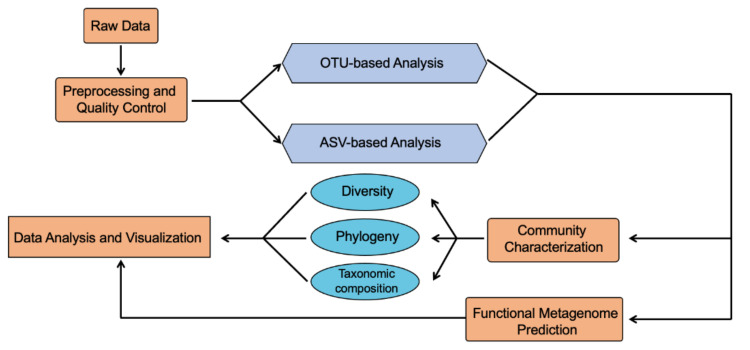
16S rRNA sequencing data analysis pipeline.

16S ribosomal RNA subunit gene contains both regions that are conserved throughout bacterial species and hypervariable regions that are unique for specific genera. 16S rRNA sequencing has been widely used to characterize the bacterial community, which utilizes PCR to target and amplify portions of the hypervariable regions (V1–V9) of the bacterial 16S ribosomal RNA subunit gene. Various bioinformatics tools have been developed in the last decade to analyze the 16S rRNA sequencing data, with most of them containing three core steps, including data preprocessing and quality control, taxonomic assignment, and community characterization (Figure 2). Quality control is the first step in the analysis pipeline, which includes quality checking, adapter removal, filtering and trimming to remove artifacts, low-quality and contaminant sequencing reads resulting from sample impurities or inadequate samples preparation steps [16]. Many quality control software packages use PHRED algorithm score to assess the base quality [17].

The taxonomic assignment is a key step in the 16S rRNA sequencing data analysis pipeline. Currently, there are two different strategies to perform this analysis: operational taxonomic unit (OTU)-based analysis and amplicon sequence variant (ASV)-based analysis. OTUs are determined by the sequence similarity. Reads are considered as the same OTU when their sequence similarity reaches a predefined similarity threshold, most commonly 97% [18]. Generally, an OTU-based analysis first clusters sequences into different OTUs and then performs taxonomic assignment. Many OTU-based methods have been developed, such as UCLUST [19], UPARSE [20], CD-HIT [21], hc-OTU [22], ESPRIT [23], ESPRIT-TREE [24]. On the other hand, an ASV-based analysis does not resolve sequence variants by an arbitrary dissimilarity threshold as used in the OTU-based analysis. Instead, ASV-based methods utilize a denoising approach to infer the biological sequences in the sample before the introduction of amplification and sequencing errors, which allows to resolve sequences differing by as little as a single nucleotide [25]. Therefore, an ASV-based analysis is able to provide a higher-resolution taxonomic result. Several ASV-based methods have been developed, including DADA2 [26], UNOISE 2 [27], and Deblur [28]. In the following part, we will introduce three representative tools that have been successfully and widely applied in 16S analysis starting from raw sequencing data, including Quantitative Insights Into Microbial Ecology (QIIME) [29,30], Mothur [31], and DADA2 [26].

QIIME 1 [29] and its next-generation, QIIME 2 [30], are open-source bioinformatics platforms for microbial community analysis and visualizations. A typical 16S analyzing workflow in QIIME 1 is:

(1) Demultiplexing and quality filter, which assigns the multiplexed reads to each sample and filters sequences that cannot meet defined quality thresholds;
(2) Chimera detection and filter, which applies ChimeraSlayer or USEARCH 6.1 to remove chimeric sequences;
(3) OTU picking and taxonomy assignment, in which sequences will be clustered into OTUs based on their sequence similarity, and taxonomy will be assigned to each representative sequence of OTUs;
(4) Community analysis, in which the community composition, phylogenetic tree, alpha- and beta-diversity can be computed or analyzed based on OTU tables.

QIIME 2 allows third parties to contribute functionality, and many latest-generation tools are embedded into the system as QIIME 2 plugins, such as DADA2 denoising and filtering. Moreover, in addition to the command-line interface like QIIME 1, QIIME 2 provides the QIIME 2 Studio graphical user interface, which is much friendlier for end-user biologists. Comparing with most of the software, both QIIME 1 and QIIME 2 provide many interactive visualization tools that allow users to generate principal coordinate analysis (PCoA) plots, alpha rarefaction plots and taxonomic composition bar plots.

Mothur is another well-known package [31]. Mothur website provides examples for data acquired from different sequencing platforms, including Illumina, Pyrosequencing, and Sanger sequencing. For Illumina 16S data, a typical analyzing workflow includes the following steps: quality control, sequence alignment, chimera removal, assignment of sequences to OTUs, analysis of community characters including taxonomy composition and diversities. Mothur is originally designed for OTU-based analysis, but the current version of Mothur also supports ASV-based analysis, in which cleaned sequences can be assigned to ASVs and taxonomy information can be analyzed based on the ASV table. The performance of Mothur and QIIME system in 16S data analysis has been compared by many previous studies in different contexts [32,33,34]. Although several differences were found between these two tools, both Mothur and QIIME can provide reliable bacterial community information and generate comparable results in general [32,33].

DADA2 is an ASV-based analysis package that utilizes DADA2 algorithm [26], a model-based approach for correcting amplicon errors without constructing OTUs. The basic analyzing workflow in DADA2 includes the following steps: quality control which filters and trims low-quality reads; sample inference and ASV table construction in which sequence variants are inferred by DADA2 algorithm and ASVs are summarized; removal of chimeric ASVs; taxonomic assignment to generate taxonomy tables. DADA2 can resolve fine-scale variation and thus provide a more accurate analysis than other OTU-based methods. DADA2 can perform species-level analysis by matching ASVs to sequenced reference strains, while traditional OTU-based methods only can provide genus or above level taxonomic information.

Although both OTU- and ASV-based methods provide the phylogenetic information, basic 16S analysis methods generally cannot provide the functional gene composition of a bacterial community. However, phylogeny is strongly correlated with biomolecular function which thus makes it is possible to predict metagenome functional content from 16S data. Several software tools have been developed to predict the functional composition of a microbial community’s metagenome from 16S data, such as phylogenetic investigation of communities by reconstruction of unobserved states (PICRUSt) [35,36] and Tax4Fun [37].

The PICRUSt algorithm composes two steps [35]. The first is called “gene content inference”, which predicts gene content for organisms in the Greengenes phylogenetic tree by using existing annotations of gene content and 16S copy number from sequenced bacterial and archaeal genomes in the IMG database. This step is pre-calculated and thus users are not required to do it in data analysis. The second step is “metagenome inference”, in which the functional gene family counts as well as the abundance of functional pathways for each sample will be predicted and summarized based on the input OTU table. The input OTU table could be generated by other 16S analyzing software, such as QIIME and Mothur. PICRUSt2 [36] is the optimized version of PICRUSt. In addition to the updated and larger database of gene families and reference genomes, PICRUSt2 is compatible with ASV-based 16S analysis. Its input file could either be an OTU table or an ASV table, while PICRUSt input is restricted to OTU tables. Now, PICRUSt2 is embedded in QIIME 2 system as a QIIME 2 plugin [30].

The R package, Tax4Fun [37], also predicts the functional capabilities of microbial communities based on 16S data but adopts a different strategy than PICRUSt. Tax4Fun predicts the metagenome functional content by the nearest neighbor identification based on a minimum 16S rRNA sequence similarity, while PICRUSt performs this by analyzing the topology of the Greengenes phylogenetic tree as described above. The input of Tax4Fun could be the OTU table obtained through QIIME analysis (against the SILVA database) or from the analysis in SILVAngs web server. The functional capabilities of the inputted microbial community are predicted using the precomputed reference profiles of the KEGG organisms. A recent study has indicated that the application of PICRUSt, PICRUSt2, and Tax4Fun on non-human and environmental samples is limited by their default databases [38]. Tax4Fun2 [39] is the updated version of Tax4Fun. Compared with the old version, Tax4Fun2 allows users to build their own reference data sets, which may enhance the accuracy and robustness of predicted functional profiles by utilizing user-defined, habitat-specific metagenome databases. Moreover, Tax4Fun2 also can be used to calculate functional gene redundancies based on 16S data.

There are some other tools that have been developed for estimating the functional capacity of a microbial community based on 16S sequencing data, such as Piphillin [40] and Vikodak [41], and each of them has some distinct features. Whole metagenome sequencing is more expensive than 16S amplicon sequencing. Therefore, functional prediction of microbial community based on 16S data will be used more frequently, in part due to substantial improvement of the accuracy of these bioinformatics tools. In addition to the tools for one or a few specific utilizations in 16S data analysis, some platforms embed various different individual tools, such as the Galaxy server (The Huttenhower Lab; https://huttenhower.sph.harvard.edu/galaxy/), MicrobiomeAnalyst (https://www.microbiomeanalyst.ca/), as well as QIIME 2 (https://qiime2.org/). These platforms allow users to perform a more comprehensive 16S analysis using a single platform.

The gut microbiome data sets are compositional, sparse and high-dimensional, which makes identifying differentially abundant microbial taxa between communities challenging. Widely used software tools optimized for statistical analysis of the microbiome data analysis includes LEfSe, MaAsLin2, etc. LEfSe discover biomarker by way of class comparison, biological consistency tests and estimation of effect size [42]. MaAsLin2 relies on general linear models to accommodate and determine multivariable association between microbial data and phenotypes, which offers a variety of methods for data normalization and transformation [43]. SparCC [44], SPEIC-EASI [45] address the compositional problem by assuming that few species are correlated, and BAnOCC [46] makes no assumptions about the microbial data. Ilr (isometric log ratio transform) is another approach controlling for false positives by testing for changes in log ratios between abundances, which does not assume few species are correlated [15]. Machine learning approaches, such as random forest, have also been applied to gut microbiome data to separate samples based on their categories, which requires a relatively larger sample size to train the model.

## 3. 18S rRNA Amplicon Sequencing and Internal Transcribed Spacer (ITS) Sequencing

Previously, researchers have mainly focused on studying the bacterial community in the gut microbiome because bacteria constitute a majority part of the gut microbiome [1,47], but recently more studies are analyzing the fungal community. The human mycobiome diversity is relatively low compared with bacterial communities and is dominated by yeast such as *Candida*, *Saccharomyces* and *Malassezia* [48]. Dysbiosis of intestinal fungi has been observed in various diseases, such as alcohol-associated liver disease [5,49], hepatitis B [6], inflammatory bowel disease [9,50,51,52], colorectal cancer [13,53], autism spectrum disorders [54], Parkinson’s disease [55].

When it comes to molecular identification of fungi, amplicon sequencing based on 18S rRNA and ITS are the most widely used methods, both of which use PCR to amplify the DNA with a specific primer, and after sequence processing, sequence analyzing, and comparing the resulting ITS sequence with the known database, the species of fungi can be identified [56,57]. 18S rRNA is a basic component of fungal cells comprising both conserved and hypervariable regions. Similar to 16S rRNA, 18S rRNA gene has nine hypervariable regions. Another commonly used barcoding marker in eukaryotic phylogenetic studies is ITS region, a 500–700 base pair (bp) nuclear ribosomal DNA sequence [56,58]. The ITS region is further separated into two regions: ITS1 (between 18S and 5.8S) and ITS2 (between 5.8S and 28S), where ITS2 is less taxonomically biased than ITS1 [56,59].

Comparing with ITS sequencing, one advantage of 18S rRNA sequencing is that it allows alignment across taxa above species level. ITS sequencing is not able to do so because of its lack of reference sequences. However, this is also a drawback for 18S rRNA sequencing because for some species, 18S rRNA sequencing can only provide information regarding taxonomic levels above species. Whereas ITS sequencing can provide lower-level information at species and subspecies levels because there is more variation in the ITS1 and ITS2 regions than 18S rRNA regions. 18S rRNA sequencing has a relatively large set of references, however, various lengths of 18S rRNA hinders the alignment of all the different regions across taxa [60,61,62,63]. ITS has a high PCR success rate and a better probability of successful fungi identification with a broader range than all other DNA regions [58]. In terms of application, ITS sequencing focuses more on studying the intraspecific genetic diversity of fungi because ITS is more variable, and 18S rRNA emphasis is more on fungi’s phylogenetic classification studies [56]. One way to provide more comprehensive classification of fungi is the combination of 18S rDNA and ITS sequencing, such as 5.8S-ITS2 [64].

The ITS and 18S rRNA amplicon sequencing analysis pipeline is similar to the 16S rRNA sequencing pipeline. Some software packages can be used for both bacterial and fungal amplicon sequencing data, such as QIIME, SSU-ALIGN [65], LotuS 2 [66], MICCA [67], and PEMA [68]. In addition, some software packages are designed only for ITS data, such as ITScan [69], ITSx [70], ITSxpress [71] and Mycofier [72]. Commonly used databases for fungi analysis include UNITE [73], ITSoneDB [74] and EukRef [75].

## 4. Shotgun Metagenomic and Metatranscriptomic Sequencing

While amplicon-based sequencing methods oftentimes only target a single gene, shotgun metagenomic sequencing is capable of random sequencing the sample’s entire metagenome without a specific primer, which alleviates biases from primer choices. Compared with marker gene-based community profiling, shotgun metagenomic sequencing adds a detailed layer to the taxonomic characterization of the community by providing information on the gene composition and the functional capacity of the gut microbiome, although it is costlier and more time-consuming than marker gene amplification. With the ability to detect organisms from all domain of life, shotgun metagenomic sequencing still represents the most effective and comprehensive approach for obtaining both structural and functional data. The gene composition can also be used to formulate putative functional pathways. Shotgun metagenomic sequencing has been applied to study the functional changes of the gut microbiome in various diseases, such as inflammatory bowel disease [76], irritable bowel syndrome [77], alcohol-associated liver disease [78,79], nonalcoholic fatty liver disease [80,81], hepatic steatosis [82], Crohn’s disease [83,84], melanoma [85], Parkinson’s disease [86], high blood pressure [87], and pulmonary tuberculosis [88].

**Figure 3:**
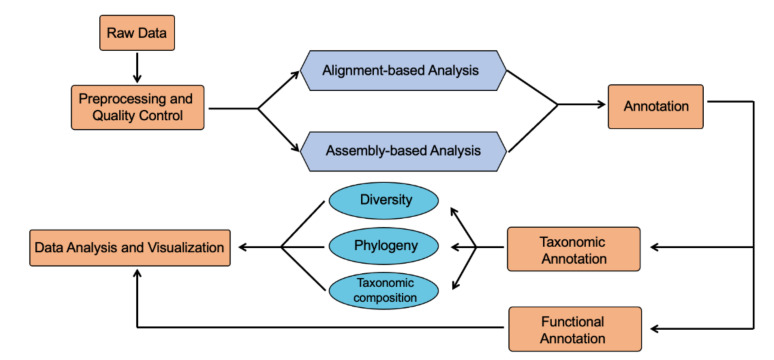
Shotgun metagenomic sequencing data analysis pipeline.

The process of shotgun metagenomic sequencing can be summarized as following: sample collection and storage, nucleic acid extraction, metagenomic library preparation, quality control, and data analysis. Quality control is the first step in the shotgun metagenomic analysis pipeline (Figure 3), which involves different tools such as Trimmomatic [89], Ktrim [90], Cutadapt [91], MultiQC [92]. The resulting high-quality reads can be either mapped to reference genomes or assembled with assembly tools. Thus, shotgun metagenomic sequencing analysis generally can be categorized into two approaches: alignment-based approach and assembly-based approach. It is often recommended to use both approaches in combination to get the most accurate results [93,94].

The alignment-based approach identifies sequencing reads’ taxonomy and functional profile through mapping the reads to known microbial reference genomes or searching against databases of characterized protein families by different mappers, such as Bowtie2 [95], DIAMOND [96], BBMap [97], etc. Different marker gene database and protein encoding gene databases are available for taxonomic and functional annotation, such as Kyoto Encyclopedia of genes and genomes (KEGG) [98], protein family annotations (PFAM) [99], gene ontologies (GO) [100], clusters of orthologous groups (COG) [101], evolutionary genealogy of genes: Non-supervised Orthologous Groups (eggNOG) [102] and UniProt Reference Clusters (UniRef) [103].

The assembly-based approach reconstructs multiple genomes even if some are yet unknown. This approach depends heavily on genome coverage. Assembly-based approach assembles short reads into contigs, which allow for multiple sequence alignment of reads relative to the consensus sequence, and then groups contigs into scaffolds, which list the order and orientation of the contigs and the size of gaps between contigs. An important parameter to assess the quality of genome assemblies is N50, which refers to the smallest contig size in a set of contigs that represents at least 50% of the assembly [104]. Metagenomic assembler generally use graph-based approaches, such as the overlap-layout-consensus and de Bruijin graph to assemble longer and shorter reads, respectively. Due to short sequence reads produced by popular sequencing platforms, de Bruijin graph-based assemblers are widely used, such as Meta-IDBA [105], IDBA-UD [106], MetaVelvet [107] and MegaHit [108], etc. The metagenome assemblers are either based on reference genome for annotation of microorganisms or based on de novo assembly which discover and reconstruct genomes without consulting databases and makes gene prediction more reliable. Generally, in the de novo assembly, metagenomic sequences are divided into pre-defined segments of size k (k-mers) which are over-lapped to form a network of overlapping paths and then form the contigs interactively [109], which is considered as the basis of de Bruijin graphs for short reads assembly [104].

The quality of assembly can be assessed by tools such as MetaQUAST [110]. The assembled genomes can be annotated through the gene family identification system in databases. Metagenomic sequence reads can also be mapped to the assembled genomes to estimate their abundance. There are some automated pipelines which integrate different steps into one convenient package, such as MEtaGenome Analyzer (MEGAN) [111], Metagenomic Phylogenetic Analysis (MetaPhlAn) [112], the HMP Unified Metabolic Analysis Network (HUMAnN2) [113], and some online servers such as Metagenomics RAST server (MG-RAST) [114], Integrated Microbial Genomes and Microbiomes (IMG/M) [115] and JCVI Metagenomics Reports (METAREP) [116], which provide an end-to-end solution. Sometimes multiple metagenomic analysis methods may produce variable results even if the same databases are used. Standardization of data processing and analysis is warranted to enable further integration of shotgun metagenomic analysis into the gut microbiome research to enhance the reproducibility and application of the analysis into clinical practice.

Although metagenomics provides access to microbial gene and genome composition and pathways, it has limited roles in revealing the gene expression in the microbial community. Shotgun metagenomic sequencing is performed on genomic DNA isolated from the biological samples; however, it is hard to distinguish whether this DNA comes from viable or dead cells or whether the genes are expressed under given conditions. Instead, metatranscriptomic sequencing allows scientists to identify whether a microbe is an active member of the microbiome or not, and to identify actively expressed genes in the microbial community to get a deeper understanding of the activity of the gene of interest. Metatranscriptomics complement shotgun metagenomics by elucidating what gens are actively transcribed from a potential repertoire of annotated genes as revealed by shotgun metagenomic analysis. Metatranscriptomic sequencing analysis has been used to study microbial RNA-based regulation and expressed biological signatures in several diseases such as inflammatory bowel disease [117] and rheumatoid arthritis [118]. It provides a snapshot of the gene expression profile under specific conditions and at a given moment, instead of its potential as inferred from DNA-based shotgun metagenomic analysis.

The construction of metatranscriptomic library starts with the isolation of total RNA and removal of host RNA contaminations which can occur to various degrees as well as removal of mRNA with probes targeting certain rRNA regions, followed by cDNA synthesis, adapter ligation and end repair. After that similar to the process of constructing shotgun metagenomic library, cDNA ends are repaired and adapters are ligated, followed by library cleanup, amplification and quantification, and the library is then sequenced on the sequencing platform. Due to the unstable nature and short half-life time, RNA isolation becomes the most difficult task, especially from some biological samples such as feces. The isolation process must be carefully carried out to avoid RNA degradation by contaminated ribonucleases, and multiple approaches specific to different cell types have been developed [119,120,121,122].

Similar to shotgun metagenomic analysis, comprehensive data analysis suites such as HUMAnN2 and MG-RAST also provide an end-to-end solution for metatranscriptomic analysis, which are combinations of multiple specialized tools, such as Trimmomatic for quality control, Bowtie for mapping, CuffDuff [123] for differential gene expression, etc. As always, quality control is the first step for metatranscriptomic analysis. An essential process in quality control step is to filter out non-mRNA reads, in addition to trimming of low-quality reads and host reads. The resulting good quality reads are used for the following analysis which are categorized into alignment-based approach and assembly-based approach. Alignment-based approach maps the sequencing reads to reference database. With assembly-based approach, the sequenced reads are first assembled into contigs, scaffolds, and then mapped to reference genomes. The assembly step is computationally challenging, which requires deeper sequencing depth and higher quality sequencing reads. The assembled transcripts are annotated through software such as Blast2GO [124] to align against protein databases, followed by normalization and calculation of relative gene expression levels and statistical analysis.

## 5. Viromic Sequencing

Viruses are key constituents of microbial communities which contribute to their evolution and homeostasis. Viromic sequencing has been used to study the intestinal viruses in different diseases, including type 1 diabetes [8], inflammatory bowel disease [10,125], alcohol-associated liver disease [126], non-alcoholic fatty liver disease [127], colorectal cancer [128,129], human immunodeficiency virus [130], and autoimmune diseases [11]. Because of the highly diverse nature of viruses and the lack of universal marker genes, it is difficult to use amplicon-based approach to amplify them with universal markers. Instead, shotgun metagenomic sequencing approaches can be used to characterize viruses and identify novel viruses.

Although in most environment, viruses outnumber microbial cells 10:1, viral DNA only represents 0.1% of the total DNA in a microbial community. Isolation of viral particles is the initial step in viromic sequencing, which is necessary to obtain a deep sequence coverage of viruses in the human gut microbiome, followed by viral particle purification. Large particles in the fecal samples, such as undigested or partially digested food fragments and microbial cells, are generally removed by serial filtration steps with osmotic neutral buffer or by ultracentrifugation with cesium chloride density gradient. The next step is nucleic acid extraction, during which the nucleic acid of the virus must first be isolated so that all the non-viral origin fractions are removed. DNAase and RNAase are usually used to remove the non-encapsulated nucleic acids. Depending on the type of viruses being studied, the library preparation protocol also varies. For example, bacteriophages are parasitic, special steps are required when isolating the DNA. For RNA virus, due to its unstable nature, reverse transcriptase to cDNA is required. In addition, virome contains active and silent fractions. For studying both the active and silent fraction of the virome, total nucleic acid isolation is needed [131]. For the active fraction of the virome, it is often required to use a filter, chemical precipitation or centrifugation to isolate the virus DNA.

The initial analysis of the sequences obtained after DNA sequencing is also quality control, which includes filtering of bad quality reads, decontamination of 16S rRNA, 18S rRNA and human sequence reads. Viruses have higher homology to prokaryotic or eukaryotic genes, therefore filtering of bad quality sequences is a key step in the viromic analysis. The resulting sequences are analyzed by either alignment-based approach or assembly approach. With alignment-based approach, different mapping algorithms are used to compare the resulting sequence reads against viral genomes and viral databases. Although the databases have expanded recently, the number of genomes deposited in the databases is far less than the sequenced virotypes and most of sequences reads lack similarity to the sequences in the databases, which are poorly annotated. The lack of sequence identity typically results in 60%–99% sequences in the viral metagenomes [132]. Due to the highly diverse nature of viruses and the lack of similarity in current existing databases, de novo assembly approaches are often used in the viromic analysis [131,133,134]. Different assemblers are used for viral metagenomic data, such as VICUNA [135]. Popular shotgun metagenome assemblers such as MetaVelvet has also been applied to viral metagenome assembly. There are some virome-specific computational pipelines available, such as Metavir [136,137] and the Viral MetaGenome Annotation Pipeline (VMGAP) [138], which generally include open reading frame (ORF)-finding algorithms to predict coding sequences, followed by comparison with different protein databases.

## 6. Conclusions

In this review, we have discussed different sequencing-based approaches, which provide useful information toward a better understanding of the role of gut microbiome in health and diseases. When studying the gut microbiome in human populations, such as healthy subjects and patients with diseases, confounding factors which could influence the gut microbiome need to be taken into consideration when analyzing the data, such as diet, medication, sex, age, life-style, etc. For example, the composition of the gut microbiome is different in infants, adults or elderly and certain discrete age range should be considered when analyzing the gut microbiota. Stool samples are often used when assessing the gut microbiome as a non-invasive approach. It is noteworthy that fecal microbiome and mucosal-associated microbiome clustered differently [139].

A list of examples of widely used tools are summarized in Table 1. For amplicon-based sequencing approaches, including the 16S rRNA sequencing, 18S rRNA sequencing, ITS sequencing, selection of target region and design of PCR primers must be performed carefully due to the primer biases. Currently, there is no agreement as to the optimal regions to be amplified, and most of the time, it is a balance between amplifying a determinative region and characterizing bacteria or fungi more broadly. For shotgun metagenomic sequencing and metatranscriptomic approaches, the turn-around time and costs need to be reduced to be introduced into clinical practice. The integration of various sequencing approaches each contribute a single piece towards a complex and large puzzle of the gut microbiome and the value of an integrative approach is greater than the sum of each part. In addition to sequencing based approaches, other -omics approaches such as metaproteomics and metabolomics complement the sequencing data, contributing to the understanding of the function and complex pathways in the gut microbial community. The global integrated approach is of great value to enable better understanding of the function of gut microbiome and move from a descriptive study to causal contributions, however, the budget and sample availability need to be taken into consideration for the integrative approach to be introduced into clinical practice.

## Author Contributions

Writing—original draft preparation, B.G., L.C., Y.Z., X.S., P.T.; writing—review and editing, J.Y., N.G., B.L., W.S., B.S.; supervision, B.S.; funding acquisition, B.S. All authors have read and agreed to the published version of the manuscript.

## Figures and Tables

**Table 1:** Examples of widely used tools to perform next generation sequencing data analysis for the gut microbiome studies.

Software	Short Description	Ref.
16S rRNA, 18S rRNA and ITS sequencing data analysis		
UCLUST/ UPARSE	UCLUST is an OTU-based clustering method. It employs USEARCH, and UPARSE is a subroutine of USEARCH which constructs OTUs de novo from next-generation reads. The general pipeline procedure of UPARSE is reads filtering, trimming, and then clustering and chimera filtering simultaneously. Pros: Able to perform de novo, closed-reference, and open-reference clustering. Cons: May filter out too many reads and result in inaccuracy of estimating the least abundant species.	[19,20]
CD-HIT	CD-HIT is one of the most used OTU-based clustering tool to decrease redundancy of sequence and improve the performance of other analysis. Pros: Uses novel parallelization strategy to achieve fast runtime; can handle extremely large databases. Cons: Diminished clustering accuracy.	[21]
Hc-OTU	Hc-OTU is an OTU-based clustering method for 16S rRNA sequence, employs homopolymer compaction and k-mer profiling. Pros: High accuracy. 7,000 times faster than MOTHUR and about six times faster than ESPRIT-TREE, while remaining the same accuracy level as MOTHUR. Supports user-specified k-mer distance threshold parameter value. Cons: Its worst-case computational complexity run time is O(n^2^), while UCLUST and CD-HIT are faster than hc-OTU with run time of O(n^1.2^).	[22]
ESPRIT	ESPRIT is an OTU-based hierarchical clustering method consisting of quality filtering, computing pairwise distance, hierarchical clustering and estimate with statistical interference. There are two version of ESPRIT, one for personal computer (small/medium size data) and one for computer clusters (large size data). Pros: Able to perform analysis on various size of data. Cons: Slow time O(n^2^) and space complexity.	[23]
ESPRIT-Tree	ESPRIT-Tree is an OTU-based online-learning-based hierarchical clustering method. ESPRIT-TREE improves on previous ESPRIT algorithm and uses a pseudometric-based partition tree. Pros: Improved runtime from ESPRIT: O(n^1.17^); relatively high accuracy. Cons: In terms of computational efficiency, UCLUST performs better than ESPRIT-Tree.	[24]
DADA2	DADA2 is an ASV-based analysis pipeline for modeling and error-correcting Illumina sequence reads. Pros: High accuracy: able to resolve single nucleotide biological differences. Can perform species-level analysis. Runtime scales linearly as sample number increase, and reasonable memory requirements. Cons: Comparably slow denoising algorithm than UPARSE.	[26]
UNOISE2	UNOISE 2 is an ASV-based tool for denoising (error-correcting) Illumina sequence reads. It is improved from UNOISE and clusters unique reads in the sequence. Pros: Higher accuracy and speed than DADA2. Cons: Does not use quality scores.	[27]
Deblur	Deblur is an ASV-based denoising tool, which uses error profiles to obtain putative error-free sequences. It operates independently on each sample. Pros: Able to obtain single-nucleotide resolution, faster than DADA2, better memory efficiency than DADA2 and UNOISE 2. Better sensitivity and specificity. Cons: Slower than UNOISE 2, limited by read length and sample sequences’ diversity.	[28]
QIIME/ QIIME2	QIIME and QIIME2 are bioinformatics platforms for microbial community analysis and visualizations. QIIME 2 is engineered based on QIIME and replaced QIIME. QIIME2 use existing bioinformatics tools as subroutines, such as DADA2, deblur, etc. Pros: Have multiple interfaces, continues to grow and adapt to novel strategies. Cons: A large number of dependent programs need to be installed.	[29,30]
Mothur	Mothur is a software analyzing raw sequences and generating visualization tools to describe α and β diversity. It is a combination of multiple analytic tools for describing and comparing microbial communities. It provides examples for data acquired from different sequencing platforms. Pros: Able to perform both ASV-based and OTU-based analysis. Cons: Relatively slow runtime and space complexity.	[31]
PICRUSt/ PICRUSt2	PICRUSt is a software for predicting functional composition based solely on marker gene sequence profiles. PICRUSt2 is the improved version of PICRUSt by having a larger reference database, enhanced prediction ability and more accurate de novo amplicon tree-building. PICRUSt2: Pros: Able to identify novel discoveries. Can process 18S and ITS rRNA sequence while the original version only supports 16s rRNA sequence analysis. Cons: Can only differentiate taxa the same level as the amplified marker gene sequence. Can be problematic if the interested microbial community’s majority phyla are not yet well-characterized.	[35,36]
Tax4Fun/ Tax4Fun2	Tax4Fun is an R package for predicting functional profiles for 16S rRNA data on the basis of SILVA-labeled OUT abundances. Tax4Fun 2 is an improved version of Tax4Fun with more accurate and enhanced prediction power. Tax4Fun 2: Pros: Easy-to-use, platform-independent and highly memory-efficient. Tax4Fun2 has higher accuracies than PICRUSt and Tax4Fun. Cons: Availability of suitable reference genomes may limit Tax4Fun 2’s performance. Only supports prediction from 16S rRNA gene.	[37,39]
Piphillin	Piphillin is a web application that produces metagenome predictions based on the nearest-neighbor mappings of 16S rRNA sequences to genome. Pros: No local computational power requirements. High correlation with corresponding metagenomic data. Higher accuracy than PICRUSt2 Cons: Have high requirements on reference database. Only supports 16S rRNA gene prediction.	[40]
Vikodak/ iVikodak	Vikodak is a web service that provides functional prediction on 16S rRNA data. It contains 3 modules: Global Mapper, Inter Sample Feature Analyzer, and Local Mapper. With these 3 modules, it is able to perform functional prediction both globally and in detail and perform pair-wise comparative statistical analysis. iVikodak is an improved version of Vikodak. Pros: No local computational power requirements. No coding skill required. Allows for single pathway probing and gene quorum assumption. Cons: Only supports prediction from 16S rRNA gene.	[41]
SSU-ALIGN	SSU-ALIGN is designed primarily to align 16S and 18S small subunit ribosomal RNA, but can also be used for large subunit ribosomal RNA alignment. Pros: High sensitivity and specificity. Cons: Not capable of inferring phylogenetic trees. Computationally expensive.	[65]
LotuS2	LotuS2 is a software pipeline for 16S/18S/ITS rRNA analysis. It is able to calculate denoised, chimera-checked OTUs and construct OTU phylogenetic tree. Pros: Fast and user friendly. Able to handle a wide variety of data sizes on a personal computer. Cons: Mapping speed limited by BLAST+.	[66]
MICCA	MICCS is a command-line software for the processing of 16S rRNA gene and ITS amplicon sequencing data, from raw sequences to OTU tables, taxonomic classification and phylogenetic tree inference. Pros: Can be used effectively on sample with a large portion of uncharacterized species. Low requirements for reference database. Memory efficient. Cons: Less estimated OTUs obtained as a comprise for high consistency.	[67]
PEMA	PEMA is a software pipeline for metabarcoding analysis based on third-party tools. Its function includes read pre-processing, OTU clustering, ASV inference, taxonomy assignment, and COI marker gene analysis. Pros: Allows partial re-execution. Fast execution time. Cons: Heavyweight computation.	[68]
ITScan	ITScan is an online pipeline for fungal diversity analysis and identification based on ITS sequences. Pros: Does not require coding skills. User friendly. Cons: Requires FASTA-formatted input file.	[69]
ITSx	ITSx is a software for detection and extraction of the ITS1 and ITS2 subregions from ITS sequences for fungi and other eukaryotes. It relies on HMMER for profile hidden Markov model analysis. Pros: Has a very high proportion of true-positive extractions and a low proportion of false-positive extractions. Cons: Requires FASTA-formatted input file.	[70]
ITSxpress	ITSxpress is a software for ITS1, ITS2 or the entire ITS region trimming. It implements HMMER and BBMerge. It is designed to support the calling of exact sequence variants rather than OTUs. Pros: Fast runtime. Processes FASTQ-formatted input file.	[71]
Mycofier	Mycofier is a machine-learning-based fungal ITS1 sequence classifier at the genus level. The final model was based on ITS1 sequences from 510 fungal genera using a Naïve Bayes algorithm. Pros: Doesn’t require pairwise sequence alignment. Cons: Only analyze fungal ITS1 sequences. BLAST approach provides higher classification accuracy.	[72]
Shotgun metagenomic and metatranscriptomic sequencing data analysis		
Trimmomatic	Trimmomatic is a sequence trimmer for Illumina sequence data. It has multiple processing steps including detection and removal of adapter and other illumine-specific sequences, and quality filtering. Pros: Processes both paired end and single end data. Cons: Slower than Ktrim.	[89]
Ktrim	Ktrim provides both adapter- and quality-trimming of the sequencing data. Pros: Faster than Trimmomatic. Cons: Higher over-trimming rates than Trimmomatic.	[90]
Cutadapt	Cutadapt is a sequence trimmer which removes adapter sequences, primers and other types of unwanted sequence from high-throughput sequencing reads. Pros: Supports 454, Illumina and SOLiD (color space) data. Cons: Slow runtime.	[91]
MultiQC	MultiQC creates a summary report visualizing output from different tools across multiple samples, facilitating the identification of global trends and biases. Pros: Provides a global view instead of per-sample analysis.	[92]
Bowtie2	Bowtie2 is a software for sequence alignment to reference genome. It supports gapped, local, and paired-end alignments. The software implements full-text minute index and SIMD dynamic programming. Pros: Memory efficient. High speed, sensitivity and accuracy. Cons: Alignment with short reads remains an active challenge (<50 bp).	[95]
DIAMOND	DIAMOND is a sequence aligner for protein and translated DNA searches. It aims to determine all significant alignments for a given input. DIAMOND uses double indexing and spaced seeds. Pros: Significantly higher speed with similar sensitivity to BLASTX. Cons: Heavy memory consuming.	[96]
BBMap	BBMap is a sequence aligner that can align DNA and RNA sequencing reads from multiple platforms, including Illumina, 454, Sanger, Ion Torrent, Pac Bio, and Nanopore. BBMap needs to index a reference before mapping to it. Pros: Fast and accurate, particularly for reads with long indels or highly mutated genomes. Has no upper limit to number of contigs or genome size. Cons: The indexing phase requires FASTA format only.	[97]
Meta-IDBA	Meta-IDBA is a de novo metagenomic assembler. It first constructs de Bruijn graph and then divides graph into connected components. Pros: Provides a multiple alignment of similar contigs from different subspecies in the same species. Cons: Unable to reconstruct the contigs of each single subspecies.	[105]
IDBA-UD	IDBA-UD is a de novo single-cell and metagenomic assembler, which can assemble sequences with highly uneven depth. It is based on de Bruijn graph approach. Pros: Implements local assembly. Cons: Sequence of species with high abundance is more likely to be misidentified as repeats.	[106]
MetaVelvet	MetaVelvet is a de novo short sequence metagenome assembler. It is extended upon the Velvet assembler (single-genome and de Bruijn-graph based) to overcome the limitations of single-genome assembler. Pros: Able to reconstruct scaffold sequences including low-abundance species. Cons: Has slightly higher percentages of chimeric scaffolds.	[107]
MegaHit	MegaHit is a de novo assembler for assembling metagenomics data. It implements succinct de Bruijn graphs. Pros: Fast and memory efficient. Available in both CPU-only and GPU-accelerated versions. Cons: Relatively biased towards the assembly of low abundant genome fragments.	[108]
MetaQUAST	MetaQUAST evaluates and compares the quality of metagenome assemblies. It is improved based on QUAST. Its metagenome specific features includes: unlimited number of reference genome, species content detection, chimeric detection, and visualizations. Pros: Can be fed with multiple assemblies. Cons: Reduced precision in order to get higher time/memory efficiency.	[110]
MEGAN	MEGAN is a BLAST-based automated pipeline for taxonomic and functional analysis of metagenomic and metatranscriptomic datasets. Pros: Allows laptop analysis of large metagenomic data sets.	[111]
MetaPhlAn/ MetaPhlAn2	MetaPhlAn is an automated pipeline that profiles the microbial composition from shotgun metagenomic data at the species-level. The microbial community it can profile includes bacteria, archaea, eukaryotes and viruses. It accomplishes profiling with unique clade-specific marker genes. MetaPhlAn 2 is extended beyond the first version with enhanced metagenomic taxonomic profiling ability. Pros: Able to work with large-scale metagenome data.	[112]
HUMAnN2	HUMAnN2 is an automated pipeline designed for functional analysis of metagenomic and metatranscriptomic data at the species-level. The general process of HUMAnN2 pipeline is identification of known species, alignment of reads to pangenomes, translated search on unclassified reads, and quantification of gene families and pathways. HUMAnN2 utilizes other pipelines such as MetaPhlAn2 to perform identification of known species. Pros: High accuracy, sensitivity, speed. Cons: A large proportion of sequencing reads remain unmapped and unintegrated.	[113]
MG-RAST	MG-RAST is a web-based fully automated system for metagenomic analysis. It provides phylogenic and functional analysis. Pros: Require only 75 bp or longer for gene prediction or similarity analysis that provides taxonomic binning and functional classification. Able to handle both assembled and unassembled data. Cons: MG-RAST has been optimized for use with the Firefox browser. There are some browser-to-browser issues with visualization of certain diagrams.	[114]
IMG/M	IGM/M is a web-based pipeline that provides comparative analysis for metagenome. It provides structural and functional annotation. Prefer assembled contigs. Pros: Integrates all datasets into a single protein level abstraction. In contrast to MG-RAST, IMG/M includes more computationally expensive tools such as hidden Markov model and BLASTX. Cons: Statistical analysis tool is only available as an on-demand computation to the registered IMG users of the Expert Review IMG site.	[115]
METAREP	METAREP is a suite of web-based tools to view and compare metagenomic annotated data including both functional and taxonomical assignments. Pros: Able to handle extremely large datasets. Able to perform comparison on up to 20+ datasets simultaneously. Cons: No inbuilt annotation workflow. Users need to upload existing annotations.	[116]
CuffDiff	Cufflinks is a suite of programs that assembles transcriptomes, estimates abundance, and performs gene expression differentiations. It implements a parsimony-based algorithm. Pros: High efficiency, sensitivity and precision. Cons: Not optimized for metatranscriptomics analysis.	[123]
Blast2GO	Blast2Go is a Blast-based software that provides automatic functional annotation on DNA/protein sequences. It has multiple annotation styles that can be used for various conditions. Pros: Combines multiple annotation strategies. Strong visualization tools. Con: Not optimized for large datasets with large number of genes.	[124]
Viromic sequencing data analysis		
VICUNA	VICUNA is a de novo assembler targeting viral populations, which have high mutation rates. Its algorithm uses an overlap-layout-consensus based approach. The general process of VICUNA is trimming reads, constructing/clustering contigs, validating contigs, and then extending and merging contigs. Pros: Able to efficiently process ultra-deep sequence data. High accuracy and continuity. Cons: Limited accessibility due to its requirement of local computing power.	[135]
Metavir/ Metavir2	Metavir is a web-based pipeline specifically for viral metagenome analysis. Metavir 2 is developed based on Metavir with additional features such as new tools for assembled virome sequence analysis and new dataset comparison strategies.Pros: User-friendly interface. Able to perform analysis on both raw reads and assembled virome sequencesCons: Focuses on the compositional analysis. Functional annotation is lacking.	[136,137]
VMGAP	VMGAP is an automated pipeline for functional annotation of viral shotgun metagenomic data. It first performs a database searches and then functional assignments. Pros: Uses specialized databases. Cons: Requires local installation of several open-source packages, programs and public databases.	[138]

## Data Availability

Not applicable.
